# Patterns of Reverse Transcriptase Inhibitor Resistance Mutations in People Living with Human Immunodeficiency Virus in Libreville, Gabon

**DOI:** 10.3390/tropicalmed10080216

**Published:** 2025-07-30

**Authors:** Guy Francis Nzengui-Nzengui, Gaël Mourembou, Euloge Ibinga, Ayawa Claudine Kombila-Koumavor, Hervé M’boyis-Kamdem, Edmery Muriel Mpouho-Ntsougha, Alain Mombo-Mombo, Angélique Ndjoyi-Mbiguino

**Affiliations:** 1 Laboratoire National de Référence IST/VIH/Sida, Département de Bactériologie-Virologie, Université des Sciences de la Santé, Libreville BP 18231, Gabon; fnzenguig@gmail.com (G.F.N.-N.); gaelmourembou@yahoo.fr (G.M.); kombilaalexane@yahoo.fr (A.C.K.-K.); hervemboyis5@gmail.com (H.M.-K.); 2 Unité de Recherche en Epidémiologie de Maladie Chronique, Santé Environnementale (UREMCSE), Département d’Epidémiologie, Biostatistique et Informatique Médicale, Université des Sciences de la Santé, Libreville BP 18231, Gabon; kmarail@yahoo.fr; 3 Service de Coordination Médicale, Service de Santé Militaire (SSM), Hôpital d’Instruction des Armées d’Akanda, Libreville BP 50, Gabon; hedmery76@gmail.com; 4 Ministère de la Santé, Libreville BP 50, Gabon; alainmombomobo@yahoo.fr

**Keywords:** people living with HIV, mutations, resistance, reverse transcriptase inhibitors, Gabon

## Abstract

**Objective:** To characterize the profiles of resistance mutations to HIV reverse transcriptase inhibitors in Gabon. **Design:** Cross-sectional study conducted over 37 months, from October 2019 to October 2022, at the IST/HIV/AIDS Reference Laboratory, a reference center for the biological monitoring of people living with the human immunodeficiency virus (PWHIV) in Gabon. **Methods:** Plasma from 666 PWHIV receiving antiretroviral treatment was collected, followed by RNA extraction, amplification, and reverse transcriptase gene sequencing. Statistical analyses were performed using Stata^®^ 14.0 software (USA). **Results:** Six hundred and sixty-six (666) PWHIV plasma collected from 252 male and 414 female patients were analyzed and 1654 mutations were detected in 388 patients, including 849 (51.3%) associated with nucleoside reverse transcriptase inhibitors (NRTIs) and 805 (48.7%) with non-nucleoside reverse transcriptase inhibitors (NNRTIs). Three of the most prescribed treatment regimens were associated to the appearance of both NRTIs and NNRTIs resistance mutations: TDF + 3TC + EFV (24.02%; 160/666); TDF + FTC + EFV) (17.2%; 114/666) and AZT + 3TC + EFV (14.6%; 97/666). Additionally, stage 3 of CD4 T-lymphocyte deficiency, the higher viral load, and treatment duration are risk factors influencing the appearance of virus mutations. Also, treatment containing TDF-3TC + DTG is more protective against mutations. **Conclusions:** Drug resistance mutations are common in Gabon and compromise the efficacy of ART. Further study must search for other causes of therapeutic failure in Gabon in PWHIV.

## 1. Introduction

In 2023, the Joint United Nations Programme on HIV/AIDS (UNAIDS) reported that 39.9 million people in the world were living with the human immunodeficiency virus (PWHIV), including 38.6 million adults and 1.4 million children under the age of 15. Only 77% (30.7 million people) of them were receiving antiretroviral treatment. In the same year, the world faced 1.3 million new HIV infections and 630,000 pandemic-related deaths. In 2023, sub-Saharan Africa remained the most affected region in the world, being home to nearly two-thirds (2/3) of PWHIV, or 62% of people infected [1].

In Gabon, the WHO 2023 report estimated the number of PWHIV at 51,000, including 48,600 adults and 2400 children under the age of 15 (https://cfs.hivci.org). Decades of experience responding to HIV show that intersecting inequalities hamper progress towards ending the AIDS pandemic [2]. In 2014, UNAIDS adopted the 90-90-90 targets, which were later reviewed and approved at 95-95-95 [3]. These targets reflect the international community’s commitment to ensure that by 2030, 95% of PWHIV will know their status, 95% of PWHIV who know their status will be receiving antiretroviral therapy, and 95% of those on ART will have an undetectable viral load [4]. In the context of Gabon, 76% (39,182) of PWHIV know their serological status, but only 58% (29,835) are receiving treatment. In addition, data on patients with an undetectable viral load have not been communicated (https://cfs.hivci.org). This problem becomes even more worrying with the insufficient coverage of ART and the appearance of HIV resistance mutations to ART. The search for resistance mutations using the HIV genotyping test has been recommended by the WHO to guide policies for dispensing antiretroviral treatment [5]. This research makes it possible to identify the mutations in the HIV genome conferring resistance to one or more antiretrovirals in order to offer the patient an efficient therapeutic regimen, while reducing the risk of transmitting strains resistant to antiretrovirals [6]. Additionally, studies have shown that testing for HIV-1 genotyping prior to initiating antiretroviral therapy improves its subsequent efficacy [7,8]. In Gabon, previous studies had already reported resistance mutations in 58% of patients [9]. This proportion was 33% in Libreville in 2008, according to the work of the National STI/HIV/AIDS Reference Laboratory of the Health Sciences University (USS) of Libreville, in collaboration with the Virology Laboratory of the European Hospital Georges Pompidou [10]. In 2010–2012, the proportion was 56.7% among pregnant women according to the work of the Interdisciplinary Centre for Medical Research of Franceville (CIRMF) [11]. Another study of 219 PWHIV residing in Franceville reported resistance mutations in 21.9% of the population studied [12]. For the most part, these studies were dated, and their sample sizes were limited. However, they had been carried out in semi-rural areas [12].

Existing studies were insufficient to have a more comprehensive view of the diversity of HIV resistance mutations to antiretrovirals in Gabon. This work aimed to characterize the profiles of HIV resistance mutations on the reverse transcriptase gene in PWHIV under treatment in Gabon.

## 2. Materials and Methods

### 2.1. Type and Setting of Study

This was a cross-sectional study conducted over 37 months, from October 2019 to October 2022 at the Department of Bacteriology–Virology of the University of Health Sciences (USS), hosting the STI/HIV/AIDS Reference Laboratory, a reference center for the biological monitoring of PWHIV in Gabon.

### 2.2. Patients

All the enrolled individuals were ART-experienced (and not naïve), and the PWHIV suspected of experiencing a failure in treatment were included after signing an informed consent form. For children, consent was provided by parents or legal guardians. The non-inclusion criteria were all PWHIV who refused to participate in the study and had an inadequate sample volume. Patients were recruited during their follow-up appointments, and were referred by clinicians from outpatient treatment centers (OTC) and infectious disease departments of hospitals in Gabon, to search for resistance mutations to antiretrovirals following a suspicion of virological failure (WHO) [13].

### 2.3. Molecular Samples and Analyses

For each patient, 5 mL of blood was collected in an EDTA tube, then centrifuged at 1500 rpm for ten minutes using the centrifuge Rotorfix 32A (Darmstadt, Germany). The collected plasma was stored at −20 °C. The search for resistance mutations was carried out on all samples. Plasma HIV-1 RNAs were extracted using the QIAamp^®^ Viral RNA, Mini Kit (Qiagen, Courtaboeuf, France). Amplification of the HIV-1 reverse transcriptase (RT) gene was performed by PCR using the thermocycler GeneAmp^®^ PCR system 9700 from Applied Biosystems using the Invitrogen Platinum Taq DNA Polymerase Kit (Thermo Fisher Scientific, Waltham, MA, USA). This step enables the reverse transcription of viral RNA into complementary DNA (cDNA) using the SuperScript III One-Step RT-PCR Kit (Thermo Fisher Scientific, Waltham, MA, USA) with the MJ3/MJ4 set of primers [14]. The nested PCR was performed using the A35/NE135 primer set [14]. Amplification was revealed on a 0.5% agarose gel. Positive samples were sent to Macrogen Europe (Amsterdam, The Netherlands) for sequencing using the ABI 3730 XL DNA Analyzer [14,15]. The sequences obtained were analyzed and corrected with Chromas pro software 2.6.6 (Technelysium Pty Ltd., South Brisbane, Australia). Resistance mutation profiles on the RT gene were generated using the Stanford interpretation algorithm version 8.9-1 Consensus sequences (https://hivdb.stanford.edu).

### 2.4. Statistical Analysis

Data were collected and entered into the Excel 8.0 collection sheet. The variables, including age, sex, viral load categories, CD4 T-lymphocyte stages, antiretroviral treatment (NRTIs and NNRTIs), treatment regimens, and treatment duration, were transformed into categorical variables. Statistical analysis was performed using Stata^®^ 14.0 Statistics/Data Analysis. Categorical variables are presented as frequencies. Mutation proportions were compared using Fisher’s exact test. Risk factors for mutation occurrence were assessed using the Mantel–Haenszel chi-square test and expressed as odds ratios (OR) with 95% confidence intervals. Additionally, the categorical variable age was transformed into two modalities: children (0 to 18 years) and adults (over 18 years). The three categories of viral load were classified as undetectable viral load (VL < 50 copies/mL), suppressed but detectable viral load (50 copies/mL < VL < 1000 copies/mL), and unsuppressed viral load (VL ≥ 1000 copies/mL) according to the WHO classification in 2023. Similarly, CD4 T-cell levels were categorized according to the CDC’s 1993 staging classification: stage 1: ≥500 cells/mm^3^, stage 2: 200 to 499 cells/mm^3^, and stage 3: <200 cells/mm^3^. For all analyses, the significance threshold was set at 0.05.

## 3. Results

### 3.1. Demographic, Immunological, Virological, and Therapeutic Characteristics

A total of 666 HIV plasmas were analyzed. Two hundred and fifty-two (37.8%) were from men and 414 (62.2%) were from women, giving an M/F sex ratio of 0.6 (Table 1). The mean age was 43.65 ± 11.25 years. The median age was 43 [ranging from 38 to 51] years, with extremes of 6 and 76 years. The 30–45-year age group was the most representative (48.6%; *n* = 324), followed by the 45–60-year age group (37.2%; *n* = 248) (Table 1). The lymphocyte profile showed that patients with stage 3 immunodeficiency were in the majority (60.9%; 406/666), followed by those with stage 2 immunodeficiency (33.3%; 222/666) (Table 1). For viral load measurements, the results showed that more than half (54.7%; 364/666) of patients had a non-viral load suppressed (Table 1). In addition, many PWHIV had been on ART for between 10 and 15 years (40.7%; 271/666) (Table 1).

### 3.2. Treatment Regimens Used in Gabon

The PWHIV included in this study were on one of the nine treatment regimens selected in Gabon, five of which are presented in Table 1. The three most prescribed regimens are TDF + 3TC + EFV (24.02%; 160/666), TDF + FTC + EFV (17.2%; 114/666), and AZT + 3TC + EFV (14.6%; 97/666) (Table 1).

### 3.3. Mutation Profiles in Patients Treated with Reverse Transcriptase Inhibitors

A total of 1654 mutations were detected in 388 patients. These mutation profiles were mainly associated with NRTIs (51.3%; 849/1654). Of them, 42% (357/849) were thymidine analogue mutations (TAMs) and included mostly T215F/I/SY/Q/Y (10.8%; 87/849) and K219E/N/Q/R (8.8%; 75/849) (Table 2). Those associated with non-TAM NRTIs were predominant (58%; 492/849) and included M184I/MV/V (33.8%; 287/849), L74I/V (6.8%; 58/849), and K65KR/N/R (6.7%; 57/849) (Table 2).

Mutations associated with NNRTIs were 48.7% (805/1654) and included K103N (34.9%; 281/805), P225H (11.1%; 89/805), and A98AG/G (8.1%; 65/805) (Table 3).

### 3.4. Associations Between the Presence of a Mutation, Sex, Age, Treatment, CD4 T-Lymphocyte Counts, Viral Load, Treatment Regimen, and Treatment Duration

#### 3.4.1. Associations Between Sex, Age, and the Presence of Mutations

The results in Table 4 showed no association between sex, age groups, and the emergence of mutations associated with NRTIs. The results in Table 5 reported no association between the sex and age groups, and the emergence of NNRTI-related mutations.

#### 3.4.2. Associations Between Antiretroviral Treatment and the Presence of Mutations

NRTI resistance mutations were found in 48.8% (325/666) of PWHIV on antiretroviral therapy (ART). In addition, a link was observed between the type of treatment and the occurrence of NRTI-associated mutations. Patients on treatment were twice as likely to develop NRTI mutations (OR 2.07, 95% CI [1.26–3.40], *p* < 0.001) (Table 4). Similarly, 56.9% (379/666) of PWHIV on ART developed NNRTI mutations. Furthermore, a link was observed between the type of treatment and the occurrence of NNRTI-associated mutations. Patients on treatment were twice as likely to develop NNRTI mutations (OR 2.51, 95% CI [1.56–4.08], *p* < 0.001) (Table 5).

#### 3.4.3. Associations Between CD4 T-Lymphocyte Counts and the Presence of Mutations

The risk of NRTI mutation occurrence was found in patients with CD4 T-lymphocyte counts <200/mm^3^ (OR 4.01, 95% CI [1.88–6.83], *p* < 0.001) (Table 4). Similarly, the risk of NNRTI mutation occurrence was found in patients with CD4 T-lymphocyte counts <200/mm^3^ (Table 5).

#### 3.4.4. Associations Between Viral Load and the Presence of Mutations

Of the 666 patients, 67.6% (*n* = 450) had a detectable viral load, while 32.4% (*n* = 216) had a viral load below the detection threshold. Of the 450 patients with a detectable viral load, 86.2% (*n* = 388) had reverse transcriptase inhibitor resistance mutations, and 13.7% (*n* = 62) had no reverse transcriptase inhibitor resistance mutations. An association was also found between viral load and the occurrence of NRTI mutations, with higher viral loads corresponding to a greater risk of mutations (Table 4). Of the latter, 81% (*n* = 314) had both nucleoside and non-nucleoside reverse transcriptase inhibitor resistance mutations, 2.8% (*n* = 11) had only NRTI-associated mutations, and 16.2% (*n* = 63) had only NNRTI-associated resistance mutations. There was also an association found between viral load and the occurrence of NNRTI mutations, with higher viral loads corresponding to a greater risk of mutations (Table 5).

#### 3.4.5. Associations Between Treatment Regimens and the Occurrence of Mutations

The results in Table 1 show that three therapeutic regimens were more prescribed and the appearance of NRTI resistance mutations were observed for TDF + 3TC + EFV (11.71%; 78/666); for TDF + FTC + EFV (10.81%; 72/666) and for AZT + 3TC + EFV (9.61%; 64/666). The TDF + 3TC + DTG regimen (OR 0.03, 95% CI [0.00–0.22], *p* < 0.001) and other regimens (ABC + 3TC + EFV; ABC + FTC + NVP; TDF + 3TC + NVP; TDF + FTC + NVP) were less likely to result in NRTI mutations, offering protection against their occurrence (Table 4).

Three regimens played a role in the appearance of NNRTI-associated mutations for TDF + 3TC + EFV (14.11%; 94/666), for TDF + FTC + EFV (12.76%; 85/666), and for AZT + 3TC + EFV (10.81%; 72/666). Similarly, the TDF + 3TC + DTG regimen (OR 0.05, 95% CI [0.01–0.23], *p* < 0.001) and other regimens (ABC + 3TC + EFV; ABC + FTC + NVP; TDF + 3TC + NVP; TDF + FTC + NVP) were less likely to result in NNRTI mutations, offering protection against their occurrence (Table 5).

In our study, an inverse association was observed between unsuppressed viral load and a low number of resistance mutations in PWHIV receiving a dolutegravir-based regimen. DTG would have a protective role. Because the high rate of resistance mutations was found in the reverse transcriptase gene, other treatment regimens likely require stopping ART. Our results support those provided in a 2023 WHO report, which found that few countries reported people not achieving viral suppression while receiving DTG-containing ART. However, these percentages fall short of those found in the same report, where results from some surveys estimated DTG resistance levels varied between 3.9% to 8.6%, with levels as high as 19.6% observed in people receiving prior treatment who switched to DTG-containing ART while having a high HIV viral load (https://www.who.int/publications/i/item/9789240086319) on 13 January 2023.

#### 3.4.6. Associations Between Duration of Antiretroviral Treatment and Presence of Mutations

Most PWHIV had received treatment. Furthermore, there was an association between treatment duration and the emergence of NRTI-associated mutations; the longer the treatment duration, the higher the risk of mutations (Table 4). Similarly, there was an association between treatment duration and the emergence of NNRTI-associated mutations; the longer the treatment duration, the higher the risk of mutations (Table 5)

## 4. Discussion

Drug resistance mutations are a major obstacle to the success of antiretroviral treatment in PWHIV. This study, aiming to characterize resistance mutation profiles to reverse transcriptase inhibitors in Gabonese PWHIV, revealed that 62.2% of patients were female. These data concurred with those previously reported in Gabon and the Democratic Republic of Congo (DRC), pointing towards the predominance of females [16,17]. In 2022, the WHO estimated that females of all ages accounted for around 59% of PWHIV in sub-Saharan Africa [2]. The median age of our study population was 43 years, meaning that HIV infection predominantly affects a sexually active population, as was also found in the DRC and Burkina Faso [17,18]. These results corroborate those obtained in a study carried out in Nigeria in 2019, where the 41–50 age group represented 31.2% of PWHIV [19].

There were more patients with profound immune deficiency, followed by those with moderate immune deficiency. These results show the levels of immunological failure, which may be explained by problems of immune reconstitution caused by probable undiagnosed opportunistic infections, or the discovery of serological status at an advanced stage of HIV infection. A weak thymus or defective bone marrow function could be involved in poor immune reconstitution [20].

The majority of patients were in major virological failure, followed by those in moderate virological failure. This suggests that wild-type virus replication continues due to the malabsorption of ART, non-adherence to ART, or discontinuation of treatment by PWHIV. Furthermore, these results show a strong association between detectable viral load and the appearance of resistance mutations to reverse transcriptase inhibitors. In our study, it was observed that the majority of patients had been on ART for between ten and 15 years. The first cause could be the absence of regular virological monitoring, enabling resistant mutants to be undetected and molecules to change rapidly. Other causes could be the appearance in patients of adverse drug effects, and the concomitant use of drugs not prescribed by doctors (parallel supply circuit). Additionally, it was previously reported that the high proportion of drug resistance mutations in patients with viral load failure, delaying the switch to second-line treatment, would increase the risk of spread of resistant strains, failure of the second-line program, increased mortality, and decreased CD4 counts. Duration of antiretroviral therapy, initial treatment regimen, and WHO clinical stages were associated with resistance mutation [21,22,23]. DTG has recently been introduced into the new national first-line treatment guidelines in Gabon, following a WHO recommendation, and is proving effective (https://www.who.int/fr/news/item/22-07-2019-who-recommends-dolutegravir-as-preferred-hiv-treatment-option-in-all-populations) on 5 January 2023. At this stage of its use, it is possible that resistance mutations to this molecule are rare, which would represent the protective character of this inhibitor against the occurrence of resistance mutations WHO recommends DTG-based ART as the preferred first- and second-line treatment. As part of the scale-up and maintenance of populations on ART, WHO recommends routinely implementing HIV drug resistance surveillance for DTG-based ART roll-out to effectively manage and prevent potential resistance. Given the evolving science on what would be considered concerning population levels of DTG-resistant HIV, WHO does not currently suggest thresholds of DTG resistance that would require specific actions at the country level (https://www.who.int/publications/i/item/9789240086319) on 13 January 2023.

The TDF + 3TC + EFV regimen was most widely prescribed, followed by TDF + FTC + EFV and AZT + 3TC + EFV, as previously reported [16].

A total of 1654 ART-resistant mutations were identified in 388 PWHIV in this study, including 51.3% NRTI-associated mutations. These data are lower than those observed in Zambia, where 81% of mutations were reported to be associated with NRTIs [24]. This difference could be due to the fact that this study involved 273 patients, whereas ours was carried out on 666 patients. Our results are close to those obtained in 2019 in Uganda (58.8%) [25]. The main mutation profiles associated with NRTIs were non-TAMs (58%), especially M184I/MV/V conferring resistance to Lamivudine (3TC) and Emtricitabine (FTC). These results are higher than those obtained in Suriname, where the M184V mutation rate was 34.2%, and in Kenya (28.8%) [26,27]. They are close to the results reported in Uganda (53.6%), Senegal (55.6%), and South Africa (54%), but lower than in Zambia (81%) [25,28,29,30]. The M184V mutation occurs rapidly in cases of virological failure on 3TC/FTC and confers high levels of resistance to these ART, both in vitro and in vivo. This mutation is also associated with functional alterations in reverse transcriptase (reduced processivity, increased fidelity, hypersensitivity to other NRTIs, altered viral fitness, and slower onset of TAMs). All these reasons would explain the residual antiviral effect and clinical benefit of maintaining 3TC/FTC in combination with other ART, despite the presence of the M184V mutation [31]. M184V/I mutations are selected by 3TC/FTC and reduce sensitivity to these drugs [32]. The M184I mutation generally appears before M184V because it results from a more common HIV-1 nucleotide substitution [32]. This fact corroborates the percentages of therapeutic failure observed in our study with regimens including 3TC/FTC (AZT + 3TC + EFV, TDF + 3TC + EFV, and TDF + FTC + EFV) and data reported in Zambia in 2020, highlighting the frequency of M184V (81%), followed by K65R (34.5%), both associated with Tenofovir (TDF) resistance [24]. Thymidine analogue mutations (TAMs) were 42%. Our results are higher than those obtained in Senegal (29.6%) and in Zambia (32.8%) [24]. TAMs confer resistance mainly to Zidovudine (AZT), Stavudine (d4T), and other NRTIs. The resistance profiles associated with K70R, K219, D67N, and T215 found in our study were lower than those previously revealed in Zambia [24]. The T215Y and M41L minor mutations found in our study, either individually or in combination, confer intermediate resistance to AZT, ABC, and TDF. The M41L/L210W/T215Y association confers intermediate resistance to AZT and TDF, but sensitivity to ABC (https://hivdb.stanford.edu).

Additionally, 48.7% of the mutation profiles were associated with NNRTIs. These rates were lower than those obtained in Suriname (73.2%) and Zambia (65.5%) [24,26]. In our study, the most representative mutation profile was K103N (34.9%), followed by P225H (11.1%) and A98AG/G (8.1%). These results were lower than those reported by Mbange et al. in Senegal, where the most frequent NNRTI-resistant genotypes were K103N (37.04%) and A98AG (18.5%) [30]. However, they were higher than those observed in Suriname, where the K103N rate was 27.8% [26]. For resistance profiles to NNRTIs, regimens including EFV or NVP were the most affected, because K103N confers resistance to both efavirenz (EFV) and nevirapine (NVP) [24]. The codon P225H confers half the sensitivity to NVP and EFV. P225H is a non-polymorphic accessory mutation, which, in combination with K103N, results in resistance in EFV-treated individuals (https://hivdb.stanford.edu). The codon A98G, a relatively non-polymorphic accessory NNRTI resistance mutation with low percentages (0.1% to 0.5%) in untreated individuals, reduces sensitivity to NVP and EFV in treated individuals (https://hivdb.stanford.edu).

In this study, five rare mutations which were virtually unknown in Gabon were reported, including two associated with NRTIs (A62AV/V and E44A/AG/D) and three associated with NNRTIs (V108I/IV, H221Y, and K238 K/T), although the percentages are low. Their associations with specific regimens may lack statistical power due to low frequencies, reducing the reliability of conclusions about their significance. Thus, in further studies, we will put more emphasis on this, as recently reported in Istanbul, Turkey [33]. Furthermore, the A62V mutation, additional non-TAMs, occurs commonly in A6 subtype viruses but is otherwise non-polymorphic (https://hivdb.stanford.edu). It is most often selected in association with K65R and Q151M multi-nucleoside resistance mutations and T69 insertions to correct the replication deficit associated with K65R and multi-nucleoside resistance mutations (https://hivdb.stanford.edu). The E44D/A mutation, an accessory TAM, contributes to reduced sensitivity to NRTIs, mainly in combination with several other TAMs (https://hivdb.stanford.edu). V108I is a relatively non-polymorphic accessory mutation that is selected in vitro by NVP, EFV, and DOR, and reduces sensitivity to both NVP and EFV (https://hivdb.stanford.edu). DOR is designed to act against most strains of HIV that are resistant to other non-nucleoside analogues [34]. K238N/T are rare non-polymorphic mutations, selected by NVP and EFV, usually in association with K103N (https://hivdb.stanford.edu). The K103T mutation alone reduces sensitivity to NVP, but does not appear to reduce sensitivity to EFV or Etravirine (ETR). (https://hivdb.stanford.edu).

Our results show a strong link between the appearance of both NRTIs and NNRTIs resistance mutations with the AZT + 3TC + EFV/NVP and TDF + FTC + EFV regimens.

Statistical analysis showed that the duration of ART was a factor favouring the occurrence of reverse transcriptase-associated resistance mutations to NRTIs (20.87%) and NNRTIs (23.72%). It had already been reported that high levels of drug resistance and an accumulation of resistance mutations could be observed as early as 12 months on ART [34,35,36]. The longer a patient remains on an ineffective treatment regimen, the more likely ART resistance mutations are to appear and accumulate. This accumulation could compromise the efficacy of second-line treatment and increase the risk of the transmission of resistant viral strains to naive patients [36,37,38,39]. However, it has been reported that a high level of resistance can develop after a short period of antiretroviral therapy in the presence of a single mutation, as for 3TC and NNRTIs [39,40,41]. For other antiproteases (IPs) and most NRTIs (AZT, ABC, DDI, d4T), a longer treatment period and a combination of several mutations are often required to observe very high levels of resistance [41,42,43,44].

Resistance mutations to ART are common in Gabon, and pose major problems for virological monitoring, due to non-compliance with the frequency of load measurements and management of PWHIV. A preliminary search for resistance mutations associated with ART should be systematically carried out before starting treatment in Gabon. The search for ART resistance mutations should become an indispensable tool for virological monitoring of HIV-1 infection in sub-Saharan Africa, and in Gabon in particular.

## 5. Study Limitations

This analysis was a cross-sectional analysis among treatment-experienced participants (no control group). Advances in ART have not made it possible to identify resistance mutations linked to HIV infection or to minority populations among PWHIV. In addition, the sequencing method used did not make it possible to detect resistant viral subpopulations (quasi-species) lower than 20%, which underlines the interest of NGS for such studies, for better patient management.

## 6. Conclusions

Finally, this study highlights the importance of CD4 T lymphocytes and HIV-1 viral load monitoring and ART resistance mutations in order to adapt antiretroviral treatment. This work highlighted the complexity of mutation profiles associated with ART resistance in the reverse transcriptase gene in Gabon, and the treatment regimens involved. The persistence of viral replication in HIV-infected patients undergoing treatment in the absence of mutations should prompt a search for other causes of therapeutic failure, notably the plasma dosage of antiretrovirals after administration, since an insufficient concentration of antiretroviral molecules or poor absorption could partly explain therapeutic failure. As a result, high-performance liquid chromatography (HPLC) assays are urgently needed to improve virological monitoring of patients on ART.

## Figures and Tables

**Table 1:** Demographic, immunological, virological, and therapeutic characteristics.

Variables	Frequencies	Percent (%)
**Sex**		
Male	252	37.8
Female	414	62.2
**Age ranges (years)**		
[0–18]	22	3.3
[>18]	644	96.7
**TCD4 lymphocytes** (/mm^3^)		
[<200]	406	60.9
[200–499]	222	33.3
[≥500]	38	5.7
**Viral load** (copies/mL)		
[<50]	97	14.6
[50–1000]	205	30.7
[≥1000]	364	54.7
**Therapeutic regimens**		
AZT + 3TC + EFV	97	14.56
AZT + 3TC + NVP	33	4.95
TDF + 3TC + DTG	46	6.91
TDF + 3TC + EFV	160	24.02
TDF + FTC + EFV	114	17.12
Others *	216	32.43
**Treatment duration (years)**		
[<5]	29	4.4
[5–10]	188	28.2
[10–15]	271	40.7
[15–20]	142	21.3
[≥20]	36	5.4
**Total**	**666**	**100.0**

* ABC + 3TC + EFV; ABC + FTC + NVP; TDF + 3TC + NVP; TDF + FTC + NVP; ABC = Abacavir; AZT = Zidovudine; 3TC = Lamivudine; EFV = Efavirenz; NVP = Nevirapine; TDF = Tenofovir; FTC = Emtricitabine and DTG = Dolutegravir.

**Table 2:** Distribution of mutations associated with NRTI resistance.

Codons	Frequencies	Percent (%)
***Non-TAMS***	***492***	
M184I/MV/V	287	33.8
L74I/V	58	6.8
K65KR/N/R	57	6.7
K70Deletion/E/G/T	55	6.5
Y115F/FY	28	3.3
A62AV/V	7	0.8
***TAMs*** *	***336***	
T215F/I/SY/Q/Y	87	10.2
K219E/N/R/Q	75	8.8
D67DN/E/G/N/T	51	6.0
M41L	49	5.8
K70R	47	5.5
L210W	27	3.2
***Accessory TAMs***	***8***	
E44A/AG/D	8	0.9
MDR **	***13***	
T69AD/D/N	**13**	1.5
**Total**	**849**	**100.0**

* thymidine analogue mutations; ** multidrug resistant.

**Table 3:** Distribution of NNRTI-associated mutations.

Codons	Frequencies	Percent (%)
K103N	**281**	**34.9**
P225H	**89**	**11.1**
A98AG/G	**65**	**8.1**
V179D/E/T/VE/VAIT	61	7.6
V108I/IV	59	7.3
G190A/S	57	7.1
K101E/H/KE/N/P	45	5.6
E138A/G/Q	42	5.2
H221Y	33	4.1
L100G/I	32	3.9
V106I/M/VI	29	3.6
K238/KN/N/T	12	1.5
**Total**	**805**	**100.0**

**Table 4:** Risk factors for the emergence of mutations associated with nucleoside reverse transcriptase inhibitors.

Variables	NRTI Mutations		
	OR	IC_95%_	*p*-Value
**Sex**			
Male	1.00		
Female	0.83	[0.61–1.14]	0.26
**Ages ranges (years)**			
[0–18]	1.00		
[>18]	0.65	[0.27–1.54]	0.32
**Antiretroviral treatment**			
No	1.00		
Yes	2.07	[1.26–3.40]	<0.001
**CD4 T cell stages (/mm^3^)**			
>500	1.00		
[200–499]	2.25	[1.03–4.90]	0.03
[<200]	3.18	[1.49–6.78]	0.001
**Viral load (/copies/mL)**			
<50	1.00		
[50–1000]	2.22	[1.16–4.27]	0.01
[>1000]	13.86	[6.97–27.56]	<0.001
**Treatment regimens**			
AZT + 3TC + NVP	1.00		
TDF + 3TC + DTG	0.03	[0.00–0.22]	<0.001
AZT + 3TC + EFV	1.10	[0.48–2.53]	0.80
TDF + FTC + EFV	0.97	[0.43–2.19]	0.96
TDF + 3TC + EFV	0.54	[0.24–1.18]	0.12
Others *	0.38	[0.17–0.83]	0.01
**Treatment duration (years)**			
<5	1.00		
[5–10]	6.34	[2.22–18.06]	0.001
[10–15]	6.71	[2.40–18.72]	<0.001
[15–20]	7.15	[2.43–21.08]	<0.001
[>20]	9.60	[2.42–38.00]	0.001

* ABC + 3TC + EFV; ABC + FTC + NVP; TDF + 3TC + NVP; TDF + FTC + NVP.

**Table 5:** Risk factors for the emergence of mutations associated with non-nucleoside reverse transcriptase inhibitors.

Variables	NNRTI Mutations		
	OR	CI_95%_	*p*-Value
**Sex**			
Male	1.00		
Female	1.01	[0.73–1.38]	0.94
**Ages ranges (years)**			
[0–18]	1.00		
[>18]	0.44	[0.18–1.25]	0.12
**Antiretroviral treatment**			
No	1.00		
Yes	2.51	[1.56–4.08]	<0.001
**CD4 T cell stages (/mm^3^)**			
>500	1.00		
[200–499]	1.95	[0.94–4.04]	0.06
[<200]	3.21	[1.58–6.53]	<0.001
**Viral load (/copies/mL)**			
<50	1.00		
[50–1000]	1.99	[1.16–3.43]	0.01
[>1000]	8.27	[4.71–14.51]	<0.001
**Treatment regimens**			
AZT + 3TC + NVP	1.00		
TDF + 3TC + DTG	0.05	[0.01–0.23]	<0.001
AZT + 3TC + EFV	1.25	[0.52–3.00]	0.61
TDF + FTC + EFV	1.27	[0.54–3.00]	0.57
TDF + 3TC + EFV	0.61	[0.27–1.39]	0.24
Others *	0.37	[0.16–0.83]	0.01
**Treatment duration (years)**			
<5	1.00		
[5–10]	6.34	[2.22–18.06]	<0.001
[10–15]	6.71	[2.40–18.72]	<0.001
[15–20]	7.15	[2.43–21.08]	<0.001
[>20]	9.60	[2.42–38.00]	<0.001

* ABC + 3TC + EFV; ABC + FTC + NVP; TDF + 3TC + NVP; TDF + FTC + NVP.

## Data Availability

The data presented in this study are available on request from the corresponding author.
